# Setting the Stage for Branched-Chain Amino Acids Use in Neurological Pathologies: Does a Single Oral Dose Provide Hours of Elevated Systemic Levels?

**DOI:** 10.3390/diseases13030076

**Published:** 2025-03-06

**Authors:** Ezek Mathew, Nathan Jones, McKinley Dews, Dominique Neal, Anders Cohen

**Affiliations:** 1 Department of Microbiology and Immunology, The University of North Texas Health Science Center, 3500 Camp Bowie Blvd, Fort Worth, TX 76107, USA; nathanjones5@my.unthsc.edu (N.J.); kinleyguinn@gmail.com (M.D.); dominiqueneal@my.unt.edu (D.N.); 2 Department of Neurological Surgery, The Brooklyn Hospital Center, 121 DeKalb Avenue, Brooklyn, NY 11201, USA; anders@tarmaak.com

**Keywords:** branched-chain amino acids, BCAA, concussion, brain injury, traumatic brain injury

## Abstract

Background: Recent studies have demonstrated that branched-chain amino acids are neuroprotective and neurorestorative. Branched-chain amino acid supplements are now being recommended to be taken before contact sports to reduce concussions. While peaks and troughs in branched-chain amino acids have previously been reported in hospital settings, the metabolism of a single recommended dose of over-the-counter branched-chain amino acids has yet to be elucidated. Methods: We analyzed a patented branched-chain amino acid product to assess its metabolism in 10 healthy adults. Results: Over the defined time points, measured levels of branched-chain amino acids remained significantly elevated when compared to the physiological baseline. The elevations in measured plasma levels indicate that a single oral dose is a viable intake option for increasing levels of branched-chain amino acids. Conclusions: This information can be leveraged to better plan branched-chain amino acid-based treatment doses in order to treat pathologies such as brain injury.

## 1. Introduction

Branched-chain amino acids (BCAA) are essential amino acids comprising leucine, valine, and isoleucine. BCAA are involved in a wide range of physiological processes, such as metabolism and neurotransmitter production. When aiming to modulate pathologies, BCAA are known to provide substantial benefits to post-traumatic brain injury (TBI) patients. Previous studies have demonstrated a significant benefit not only in reducing but also in potentially preventing TBI sequelae [1,2,3]. In 2022, Dickerman et al. demonstrated that BCAA provided clinical and neuropathological protection when administered to mice prior to suffering moderate to severe TBI. The authors went on to demonstrate that the animals who had elevated BCAA levels in their bloodstream prior to injury and remained on BCAA after injury behaved clinically similarly to the mice who were not injured at all. At the conclusion of the study, all animals underwent neuropathological examinations. Moreover, post-mortem neuropathological examinations of the brains in the BCAA-treated group showed little to no injury; this was in stark contrast to the untreated TBI groups, which displayed neuropathological damage along with severe neurological dysfunction [2]. In 2024, a human study examined the beneficial effects of BCAA on post-concussion recovery rates in adolescents who were diagnosed with a sports concussion. The authors concluded that BCAA provided significant benefits to the athletes, enhancing their recovery [3]. Earlier animal studies investigated the administration of BCAA to mice after TBI induction, concluding that the animals’ neurological function significantly improved while on BCAA. An even more interesting finding was that animals’ neurological function would decline when the BCAA treatment was withdrawn, demonstrating a direct correlation between BCAA levels and neurological function [4].

While the beneficial effects of BCAA administration are evident throughout the literature, questions arise about the actual therapeutic levels required in humans for treatment. These questions are important for the possible prevention or reduction of TBI sequelae, especially in populations at a higher risk of TBI, such as athletes in contact sports or military personnel. Thus, based on prior studies, we set out to test a BCAA formula that was designed for the prevention and reduction of TBI sequelae. We assessed the plasma BCAA levels at baseline, 30 min after the administration of BCAA, and 3 h after the administration of a single BCAA oral dose.

In this work, we summarize the findings of this human study. After the administration of an oral dose, the levels of all three BCAA were significantly elevated for both the 30 min post-dose and 3 h post-dose timeframes.

## 2. Results

### 2.1. Tolerability

All subjects completed the study without complaints or side effects from the BCAA supplement.

### 2.2. Measured Concentration Levels

Blood was collected from participants at three time points. One blood draw occurred at 0 min (serving as the baseline value immediately before BCAA administration), 30 min post-intake, and 180 min post-intake. The concentrations for each of the 10 participants, along with the average values, are summarized in Table 1 for the amino acid leucine. Baseline measurements for leucine, in terms of mean value ± standard deviation, were 107.9 ± 21.6 μmol/L for the 10 participants. This value aligns with the baseline leucine levels reported in the literature, which normally range between 67 and 166 μmol/L for adults. The measurements of isoleucine concentrations, which normally range between 33 and 88 μmol/L, are listed in Table 2. For isoleucine, the mean blood plasma concentration value was 65.9 ± 15.1 μmol/L, which also aligns with the existing literature. Table 3 lists the plasma concentration of the amino acid valine, which normally ranges between 133 and 317 μmol/L. While the mean value of the baseline valine measurements for the 10 participants was 240.2 ± 37.5 μmol/L, there were two instances of outliers. For valine, one participant exhibited a baseline value that was below the normal range, at 130 μmol/L. Additionally, one participant exhibited a baseline value that was above the normal range for valine, measured at 323 μmol/L. However, both of these values were only modestly outside of the normal range. No other outliers were noted, and the average values for the 10 participants ranged between the normal adult values for leucine, isoleucine, and valine.

**Figure 1:**
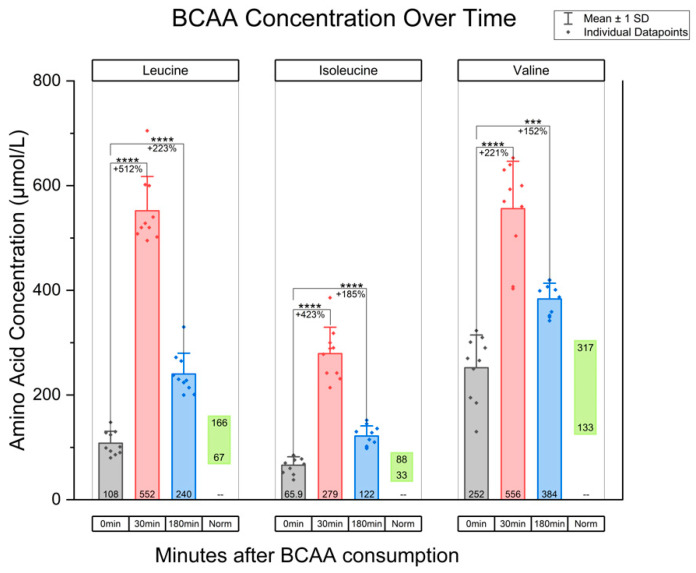
Graph of BCAA concentration over time, for the amino acids leucine, isoleucine, and valine. The 0 min time point indicates BCAA concentration at baseline. Additionally, BCAA concentration at 30 min post-oral intake and 180 min post-consumption are included. “Norm” refers to the physiological ranges of amino acids. Paired *t*-tests were used, with Bonferroni correction applied for two comparisons at α = 0.05. This resulted in a threshold for statistical significance of *p* < 0.025. Note: *** indicates *p* < 0.001; **** indicates *p* < 0.0001.

In terms of the measured plasma concentrations after the administration of the BCAA oral dose, plasma leucine concentration increased approximately five-fold from baseline levels at 30 min post-intake. This was a statistically significant increase when compared to baseline (mean difference: 444.1, 95% CI, *p* < 0.0001). At three hours post-intake, the leucine concentration remained at more than twice the average baseline level (mean difference: 131.5, 95% CI [102.9, 161.7], *p* < 0.0001). Isoleucine concentration increased to four times the baseline level at 30 min post-intake (mean difference: 213.1, 95% CI [172.6, 253.6], *p* < 0.0001) and remained at nearly double the baseline level at 3 h (mean difference: 55.7, 95% CI [36.9, 74.5], *p* < 0.0001). Lastly, valine rose approximately three-fold at 30 min (mean difference: 304.0, 95% CI [214.6, 393.4], *p* < 0.0001). The concentration trended downward but remained nearly twice the baseline level at 3 h (mean difference: 131.5, 95% CI [78.3, 184.7], *p* = 0.003). These data are summarized graphically in Figure 1. In addition, Supplementary Figure S1 superimposes lines tracking amino acid levels for each participant. Ultimately, all BCAA demonstrated a durable elevation by remaining at concentrations above normal physiological baseline at both 30 min and 3 h post-intake in these 10 individuals. Additionally, these blood plasma amino acid levels were significantly higher than baseline values.

## 3. Discussion

A key rationale for this study was to assess whether blood amino acid levels could be significantly elevated following a single oral dose and, if so, to determine the duration of this elevation. The observed increases in plasma amino acid concentrations confirmed that oral administration is an effective method for raising blood levels, reinforcing the feasibility of this delivery approach. By establishing this foundational understanding, we can now investigate the broader therapeutic implications of BCAA supplementation. Future research can focus on exploring its potential applications in various clinical contexts, such as metabolic disorders, neuroprotection, and muscle recovery, as well as optimizing dosing strategies to maximize efficacy and safety for different patient populations. In the prior literature, BCAA treatment was shown to be effective in promoting recovery in various neurological conditions, one of which is TBI [3,5,6,7]. While we did not include patients with neurological damage in this study, we sought to evaluate the plasma concentrations in healthy subjects to offer baseline values for further research. This study thus serves a foundational purpose, demonstrating that oral BCAA administration significantly elevates plasma amino acid concentrations. Indeed, this trend was observed for all three BCAA within the assessed time frame. This information is necessary from a physiological standpoint before applying BCAA treatment to patients with severe pathologies. In this study, the lack of BCAA testing on patients afflicted with TBI is a limitation. However, this work is intended as an a priori exploration, investigating BCAA’s therapeutic potential for TBI as a future objective. The potential application of BCAA in neurological injury treatment should be considered in the context of the existing literature, which highlights both its benefits and the complexities of its role in neuroprotection.

When considering the needs of particular populations, it is important to note that some risk factors cannot be attenuated. This is especially true for those with high potential for injury, such as contact sport athletes and military personnel. In addition to BCAA being used post-injury for reducing TBI symptoms, the persistent elevation of BCAA seen in this study may imply that BCAA could be used as a preventative intervention. When considered in the context of the existing literature, this study of plasma amino acid concentration supports the use of BCAA in two paradigms. BCAA can be useful in the post-injury period due to the rapid increase in plasma amino acid levels. BCAA could also be useful in pathologies such as TBI, serving a neurorestorative function as seen in prior studies [2,7,8]. Additionally, due to the persistent elevation of amino acid levels, there is potential for BCAA to be used pre-injury. In populations with a high incidence of TBI, BCAA could serve as a preventative, neuroprotective element [2].

A deeper understanding of the biological mechanisms by which BCAA influences brain function is essential for evaluating its therapeutic viability. To that end, we will discuss the mechanisms through which BCAA contributes to brain protection and recovery, particularly in the regulation of metabolic processes. The balance between essential and non-essential amino acids is a tightly controlled physiological process that is dynamically regulated in response to the metabolic demands of both the brain and muscles [9]. Leucine is the most abundant of the BCAA and is known as a very potent activator of mTOR. This amino acid is likely one of the reasons for the benefits seen when BCAA are used to treat TBI [10]. Leucine has important functional roles in the setting of TBI, as it acts as a primary buffer to the glutamate spike that occurs after TBI. Regarding nitrogen balance, leucine is responsible for almost 50% of the nitrogen donated in the brain [11]. Earlier studies attempted to provide only leucine, ignoring the utility of other branched-chain amino acids. With this single-amino-acid approach, the authors found that leucine alone induces a deleterious imbalance [9]. The ideal ratio appears to be a 2:1:1 ratio (leucine, isoleucine, and valine), which aligns with the usual composition found in animal proteins [12]. Dickerman et al. utilized this 2:1:1 ratio in their murine TBI model study, which was the first study to demonstrate the neuroprotective effects of BCAA in TBI [2].

Several points are worth mentioning with regard to the influence of BCAA levels in TBI. Firstly, it is known that TBI patients have very low serum values of BCAA upon arrival to the emergency room, post-injury. While some have suggested that these lower BCAA levels are present in TBI patients due to poor nutrition, alternate explanations do exist [2,13]. The apparent reduction in BCAA levels may be attributed to increased uptake, as the body consumes valuable proteins after injury. Yudkoff et al. proposed that leucine participates in a leucine–glutamate shuttle, which can increase activity within seconds of brain injury. Thus, if the brain is rapidly utilizing the systemic BCAA to repair neural tissue, this would lead to lower circulating levels of BCAA [11].

Secondly, prealbumin and albumin levels tend to be low in TBI patients. Considering these findings, it has been postulated that the increased blood–brain barrier permeability after TBI allows albumin and prealbumin to be redistributed. This distribution subsequently lowers the measurable levels, although some researchers assume prealbumin is low due to the poor nutritional intake in TBI patients due to neurological deficits [13,14,15]. Earlier studies analyzed the response of low prealbumin levels to BCAA by assessing chronic TBI patients with below-normal prealbumin levels in a rehabilitation hospital setting. The patients were given an oral BCAA supplement, which rapidly reversed the low prealbumin levels [16]. It was postulated that the synthesis of prealbumin requires BCAA. Thus, a patient with a TBI will prioritize the systemic pool of BCAA to repair the CNS damage. While this is a physiological response, insufficient BCAA is present in the pool to synthesize prealbumin.

The third point to note comes in the form of a more recent theory, which states the gastrointestinal microbiome is altered in TBI patients, allowing for an overgrowth of certain bacteria that inhibit the absorption of BCAA. Consequently, this phenomenon may alter growth hormone synthesis, among other essential proteins. This gastrointestinal-brain feedback loop could be key to assisting TBI patients in their chronic state [17]. While this overgrowth of bacteria would not explain the low BCAA levels in the acute phase, there are other benefits of BCAA dosing that arise in the short term. One must look back at the highly impressive studies by Aquilani et al., which demonstrated that an IV route of BCAA (which would bypass the gut microbiome) provided a highly statistically significant clinical effect in the severe TBI patients who were months outside of initial injury [5,6]. The altered gut microbiome and subsequent malabsorption of BCAA demonstrate the complexity of TBI in all phases and how TBI affects multiple organ systems [17]. The findings also further support the importance of BCAA to the brain in the acute and chronic phases of TBI [1,2,17].

The role of BCAA in neurotransmitter balance is complex, and the literature remains unclear in some respects. While BCAA serves as a nitrogen donor for glutamate synthesis, which functions as an excitatory neurotransmitter, it can also contribute to the production of gamma–aminobutyric acid (GABA), which plays an inhibitory role in neuronal transmission in the brain [18]. In the context of TBI, BCAA could modulate the levels of glutamate, which is a neurotransmitter that can contribute to excitotoxicity. In instances of neuronal injury leading to excitotoxic activity, BCAA metabolism has protective functions by allowing for the shunting of glutamate into glutamine, which is not excitotoxic. Additionally, BCAA may play a role in the export of excitotoxic glutamate via the reamination of branched-chain ketoacids [19]. However, as more research is needed in this area, this is a valuable future direction warranting further investigation.

While the results of this study provide valuable insights into the potential role of BCAA in neuroprotection, it is important to acknowledge the inherent limitations of this study. In particular, the small sample size, a characteristic of exploratory studies, presents challenges in statistical analysis and interpretation. In studies with smaller sample sizes, detecting statistically significant differences between conditions is often challenging. However, this was not the case in our study, as we observed a significant elevation in blood plasma amino acid concentrations following oral BCAA administration. While smaller sample sizes can affect statistical power and increase variability, our baseline amino acid levels do appear to align well with the various ranges that are reported in the literature for leucine, isoleucine, and valine.

A unique aspect of our study was the inclusion of an equal number of male (n = 5) and female (n = 5) participants. The existing literature suggests that BCAA metabolism differs by gender in animal models, influenced by factors such as time-dependent catabolism rates [20]. Additionally, gender may differentially influence the physiological effects of BCAA between males and females. This was demonstrated in a human study evaluating the effect of BCAA on exercise performance across various domains [21]. While a direct comparison of amino acid levels between male and female participants would have provided valuable insights, our confidentiality agreement prevented us from associating gender details with blood plasma concentration data over time. However, this remains an important avenue for future research to determine whether BCAA dosing guidelines should be adjusted based on patient gender.

Similarly, while our study included participants aged 21 to 40 years, incorporating pediatric and geriatric populations would enable a broader metabolic analysis of oral BCAA across different age groups. We were unable to attribute specific age metrics to blood plasma levels due to the same confidentiality constraints, precluding age-based comparisons after oral dosing. However, investigating the influence of age on BCAA uptake and metabolism would be a critical direction for future studies, as age-related metabolic differences may impact the efficacy and optimization of BCAA supplementation. One human study observed reductions in leucine and isoleucine levels, a trend that was correlated with age [22]. The examination of wide age ranges is also warranted due to research indicating that an individual’s age may have effects on BCAA catabolism [23].

While this study is not without limitations, future directions could also further investigate the effect of participant age by incorporating age-stratified analyses. The effect of repeat dosing of BCAA on the plasma concentration of individual amino acids is a valuable future direction, which is an investigation that is missing from the current literature. In conjunction, assessing BCAA levels beyond the 3 h timeframe after oral dosing could allow researchers to gain insights into the longer-term metabolic profile of BCAA dosing and sustained bioavailability. Additionally, the inclusion of patient populations affected by traumatic brain injury (TBI) would be a necessary phase II study. Evaluation of the efficacy of treatment’s effect on factors such as neurological disability scores could be evaluated at this stage by comparing treated and untreated controls. Considering that metabolic responses may vary under stressful conditions such as TBI, further investigation of the pathological state would provide a more comprehensive understanding of BCAA metabolism and its potential therapeutic applications, while also allowing for further research to understand if the dosing criteria need to be changed when compared to normal participants.

It would also be necessary to consider the exclusion of patients who do not have a normal BCAA metabolism. For instance, dietary restriction of BCAA is indicated for patients with metabolic diseases such as maple syrup urine disease (MSUD). Neurological derangements can be observed due to the toxic accumulation of BCAA in the bloodstream of these patients [24]. There may also be other rare metabolic disorders that alter the catabolism and accumulation of BCAA. While further research is needed to understand the implications of BCAA treatment in these patients, this will be a future consideration for our group. At this time, more research is needed to increase the clinical applicability of BCAA for the treatment of neurological conditions and tailor treatment to populations of interest. Ultimately, this study seeks to bridge the gap between preclinical studies evaluating the effects of BCAA for various use cases and clinical studies evaluating the potential of BCAA for the treatment of various neurological ailments without offering a rationalization for dosing guidelines or the evaluation of physiologically normal participants. However, this work lays the foundation for further work by sharing insights into the physiological effects of BCAA in healthy participants. This research will allow future researchers to optimize the oral dosage amount and consider the timescale of the effects.

In summary, this small human metabolic study supports the prior literature by Matsumoto et al. while adding unique discoveries to the body of knowledge [9]. These findings strongly support the notion that after a single oral BCAA dose at a 2:1:1 ratio, plasma BCAA levels remain significantly elevated above the baseline for at least 3 h. When considering the findings of this study alongside the prior literature, our data provide a foundation for optimizing BCAA dosing in human participants who may benefit from supplementation. Future research will focus on adapting these findings to develop precise dosing regimens for patients with neurological pathologies. As consistently highlighted in the literature, BCAA treatment shows promise as a potential therapeutic option across various clinical applications while exhibiting fewer severe side effects compared to conventional medications. However, further research is needed to fully elucidate the therapeutic potential of BCAA and their role in improving patient outcomes. One promising application is in the treatment of traumatic brain injury (TBI), particularly for individuals at high risk, such as contact sports athletes and military personnel. By refining the BCAA dosing strategies, this approach may offer neuroprotective benefits and improve recovery outcomes in these vulnerable populations.

## 4. Materials and Methods

### 4.1. Human Participants

This study recruited 10 healthy volunteers, evenly distributed by sex (n = 5 males and n = 5 females), aged 21 to 40 years. Although we had the age range of the individuals, along with the gender split, our confidentiality agreement did not allow us to pair blood values with demographic data. In essence, we were blinded to variables other than the blood levels of amino acids. These participants had no significant medical history, and had no previous diagnoses of inflammatory conditions, cardiovascular pathologies, metabolic diseases, chronic health issues, or other comorbid conditions. No other demographic information was collected from the participants, as this criterion was included in the study agreement. Patient information was de-identified and completely inaccessible to the investigators to maintain compliance with HIPAA regulations. The only information accessible to investigators was the serial value of amino acid concentrations, which was transmitted through a third-party provider (Labcorp, Raritan, NJ, USA), in accordance with the HIPAA regulations. All subjects were educated on the nature of this agreement and informed of the measurements available to investigators. All parties agreed to this information transfer without objection. As this was an exploratory pilot study, the primary objective was to generate preliminary data and evaluate the magnitudes of the changes in amino acid levels following BCAA dosing, rather than to test a specific hypothesis. Given this exploratory nature, a formal power analysis was not performed for this study; thus, the sample size was arbitrary.

### 4.2. BCAA Intake

The BCAA supplement examined in this paper is a 2:1:1 ratio of leucine, isoleucine, and valine by weight. Thus, each participant consumed 5 g of leucine, 2.5 g of isoleucine, and 2.5 g of valine. As a nutraceutical, these BCAA constituents were mixed into a pre-defined volume of water according to manufacturer specifications in order to allow for human consumption. This free-form BCAA supplement is named AMINOHEAL Powder, (LOT#22633), from AMINOHEAL, Dallas, TX, USA. This supplement is a patented, commercially available supplement that is mass-produced with strict adherence to current good manufacturing practice (CGMP) regulations. Participants were educated on how to consume the BCAA mixture and were observed to ensure proper intake.

### 4.3. Measurement of BCAA Plasma Levels

Consent was obtained from each of the 10 participants to draw blood three times during the duration of the study. Blood was drawn at a registered LabCorp location by professional phlebotomists. A total of 4 mL of blood was drawn from the participants immediately prior to consumption of the BCAA supplement, defined as the baseline, or 0 min time frame. After drinking the BCAA complement, 4 mL of blood was drawn from each of the participants 30 min after consumption. A final 4 mL of blood was drawn 180 min post-consumption. Blood was stored securely and transferred to a central testing location. Liquid chromatography–mass spectrometry (LC-MS) was used to quantify the amino acid concentrations in blood plasma, and results were compiled by a blinded third party.

### 4.4. Statistical Measures

To assess for significant differences between amino acid levels, the paired *t*-test was used. Comparisons were made between the amino acid levels at baseline and at 30 min after consumption. Another comparison was made between baseline amino acid levels and levels 180 min after consumption. To control the Type I error rate associated with multiple comparisons, the Bonferroni correction was applied for the two comparisons. For an initial α = 0.05, the corrected threshold for statistical significance was set at *p* < 0.025 for the two comparison time points.

## 5. Conclusions

The beneficial effects of BCAA treatment have been explored in the prior literature, demonstrating the neuroprotective and neurorestorative effects in various models. This is especially true for neurological ailments such as TBI, a condition for which there is no FDA-approved treatment. To advance therapeutic applications and harness the benefits of BCAA in human subjects, critical information on dosing guidelines and effective timing is required. Given the limited research in this area, this study aims to address these gaps by providing foundational data. Our findings demonstrate that 30 min after a single oral dose, the amino acid levels of leucine, isoleucine, and valine were significantly elevated. This effect persisted even three hours after administration, highlighting oral administration as an effective delivery method. The dual characteristics of both the rapid and sustained elevation of plasma amino acid levels is highly valuable when considering the use of BCAA for the treatment of pathologies such as TBI.

## Figures and Tables

**Table 1:** Concentrations of leucine for n = 10 subjects, measured at baseline, 30 min after oral administration, and 180 min after oral administration. Note: The normal range of leucine for adults is between 67 and 166 μmol/L.

Subject	Leucine Concentration, Baseline (μmol/L)	Leucine Concentration, 30 min Post-Dose (μmol/L)	Leucine Concentration, 180 min Post-Dose (μmol/L)
1	124	520	224
2	93	705	265
3	128	528	228
4	99	495	230
5	86	540	214
6	80	600	272
7	130	602	330
8	90	520	200
9	148	502	238
10	101	508	201
Average	107.9	552.0	240.2
Standard Error	21.6	62.2	37.5

**Table 2:** Concentrations of isoleucine for n = 10 subjects, measured at baseline, 30 min after oral administration, and 180 min after oral administration. Note: The normal range of isoleucine for adults is between 33 and 88 μmol/L.

Subject	Isoleucine Concentration, Baseline (μmol/L)	Isoleucine Concentration, 30 min Post-Dose (μmol/L)	Isoleucine Concentration, 180 min Post-Dose (μmol/L)
1	76	300	152
2	48	386	100
3	84	289	115
4	60	242	98
5	38	318	145
6	85	290	128
7	78	278	102
8	70	242	110
9	68	214	130
10	52	231	136
Average	65.9	279.0	121.6
Standard Error	15.1	47.9	18.4

**Table 3:** Concentrations of valine for n = 10 subjects, measured at baseline, 30 min after oral administration and 180 min after oral administration. Note: The normal range of valine for adults is between 133 and 317 μmol/L.

Subject	Valine Concentration, Baseline (μmol/L)	Valine Concentration, 30 min Post-Dose (μmol/L)	Valine Concentration, 180 min Post-Dose (μmol/L)
1	266	653	359
2	185	640	419
3	323	403	342
4	195	504	349
5	130	593	407
6	301	600	401
7	310	630	420
8	250	560	352
9	290	407	387
10	270	570	399
Average	252.0	556.0	383.5
Standard Error	59.5	85.9	28.7

## Data Availability

Data pertaining to the measured plasma levels of individual branched-chain amino acids are listed for each study participant in Table 1, Table 2 and Table 3.

## Supplementary Materials

The following supporting information can be downloaded at: https://www.mdpi.com/article/10.3390/diseases13030076/s1, Figure S1: Graph of BCAA concentration over time, for the amino acids leucine, isoleucine, and valine.
