# Supplementary material for: Setting the Stage for Branched-Chain Amino Acids Use in Neurological Pathologies: Does a Single Oral Dose Provide Hours of Elevated Systemic Levels?
Source: Diseases. 2025 Mar 6;13(3):76. doi: 10.3390/diseases13030076 (PMC11941354; doi:10.3390/diseases13030076)
Supplement: Supplementary file 1 [file diseases-13-00076-s001.zip › diseases-3455362-supplementary.pdf]

Setting the Stage for Branched-Chain Amino Acids Use in Neurological Pathologies: Does a Single Oral Dose Provide Hours of Elevated Systemic Levels?

Ezek Mathew [1,*], Nathan Jones [1], McKinley Dews [1], Dominique Neal [1] and Anders Cohen [2]

[1] Department of Microbiology and Immunology, The University of North Texas Health Science Center, 3500 Camp Bowie Blvd, Fort Worth, TX 76107, USA
[2] Department of Neurological Surgery, The Brooklyn Hospital Center, 121 DeKalb Avenue, Brooklyn, NY 11201, USA

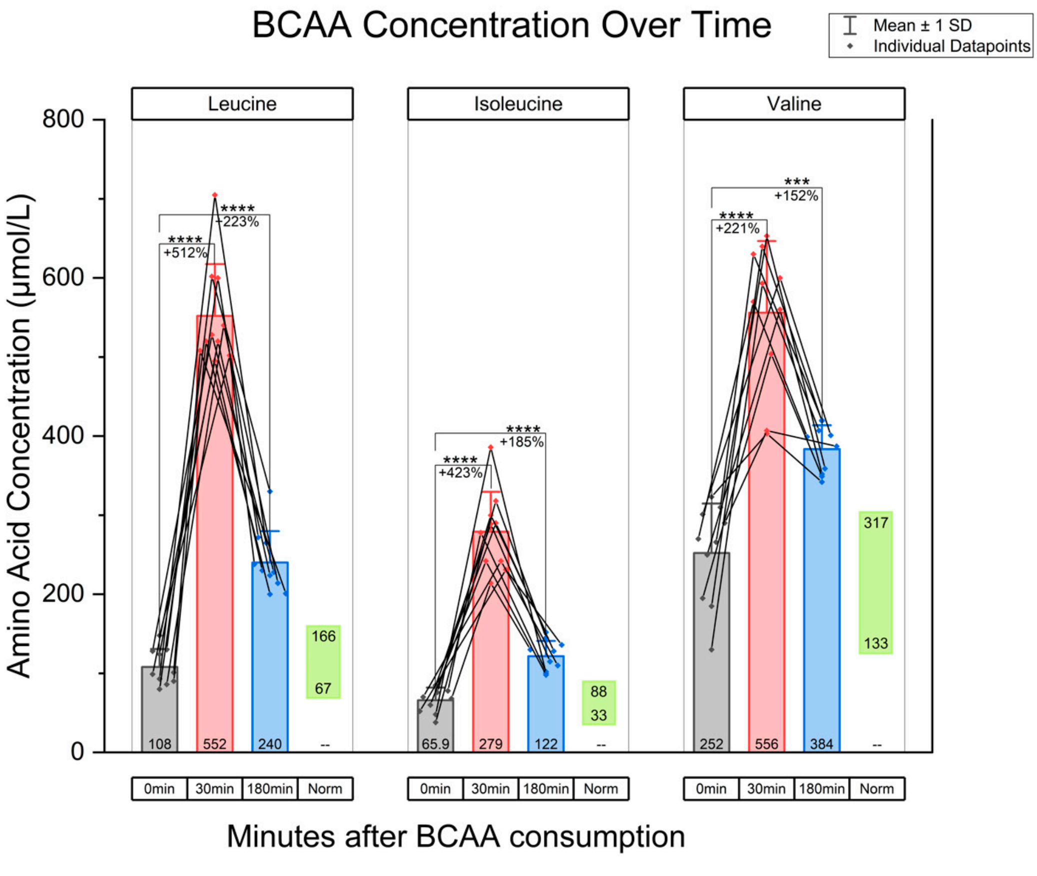

Supplementary Figure 1 (Figure S1). Graph of BCAA concentration over time, for the amino acids leucine, isoleucine, and valine. Individual lines connect the amino acid level data points pertaining to each individual participant. The 0 min time point indicates BCAA concentration at baseline. Additionally, BCAA concentration at 30 minutes post-oral intake and 180 minutes post-consumption are included. "Norm" refers to the physiological ranges of amino acids. Paired t-tests were used, with Bonferroni correction applied for two comparisons at α=0.05. This resulted in a threshold for statistical significance at $p < 0.025$. Note: *** indicates $p < 0.001$; **** indicates $p < 0.0001$.
